# Frameshift coding sequence variants in the *LPL* gene: identification of two novel events and exploration of the genotype–phenotype relationship for variants reported to date

**DOI:** 10.1186/s12944-023-01898-w

**Published:** 2023-08-11

**Authors:** Guofu Zhang, Yuepeng Hu, Qi Yang, Na Pu, Gang Li, Jingzhu Zhang, Zhihui Tong, Emmanuelle Masson, David N. Cooper, Jian-Min Chen, Weiqin Li

**Affiliations:** 1 Department of Critical Care Medicine, Nanjing Jinling Hospital, Affiliated Hospital of Medical School, Nanjing University, Nanjing, China; 2 Univ Brest, Inserm, EFS, UMR 1078, GGB, 29200 Brest, France; 3 Service de Génétique Médicale Et de Biologie de La Reproduction, CHRU Brest, 29200 Brest, France; 4 Institute of Medical Genetics, School of Medicine, Cardiff University, Cardiff, UK; 5 Institute of Critical Care Medicine, Nanjing University, Nanjing, China

**Keywords:** Aberrant splicing, Cryptic splice site, Familial chylomicronemia syndrome, Genotype–phenotype relationship, Hypertriglyceridemia-related acute pancreatitis, In-frame variant, Lipoprotein lipase, *LPL* frameshift variant, Triglyceride, Zygosity

## Abstract

**Background:**

Lipoprotein lipase (LPL) is the rate-limiting enzyme for triglyceride hydrolysis. Homozygous or compound heterozygous *LPL* variants cause autosomal recessive familial chylomicronemia syndrome (FCS), whereas simple heterozygous *LPL* variants are associated with hypertriglyceridemia (HTG) and HTG-related disorders. *LPL* frameshift coding sequence variants usually cause complete functional loss of the affected allele, thereby allowing exploration of the impact of different levels of LPL function in human disease.

**Methods:**

All exons and flanking intronic regions of *LPL* were Sanger sequenced in patients with HTG-related acute pancreatitis (HTG-AP) or HTG-AP in pregnancy. Previously reported *LPL* frameshift coding sequence variants were collated from the Human Gene Mutation Database and through PubMed keyword searching. Original reports were manually evaluated for the following information: zygosity status of the variant, plasma LPL activity of the variant carrier, disease referred for genetic analysis, patient’s age at genetic analysis, and patient’s disease history. SpliceAI was employed to predict the potential impact of collated variants on splicing.

**Results:**

Two novel rare variants were identified, and 53 known *LPL* frameshift coding sequence variants were collated. Of the 51 variants informative for zygosity, 30 were simple heterozygotes, 12 were homozygotes, and 9 were compound heterozygotes. Careful evaluation of the 55 variants with respect to their clinical and genetic data generated several interesting findings. First, we conclude that 6–7% residual LPL function could significantly delay the age of onset of FCS and reduce the prevalence of FCS-associated syndromes. Second, whereas a large majority of *LPL* frameshift coding sequence variants completely disrupt gene function through their "frameshift" nature, a small fraction of these variants may act wholly or partly as "in-frame" variants, leading to the generation of protein products with some residual LPL function. Third, we identified two candidate *LPL* frameshift coding sequence variants that may retain residual function based on genotype–phenotype correlation or SpliceAI-predicted data.

**Conclusions:**

This study reported two novel *LPL* variants and yielded new insights into the genotype–phenotype relationship as it pertains to *LPL* frameshift coding sequence variants.

**Supplementary Information:**

The online version contains supplementary material available at 10.1186/s12944-023-01898-w.

## Background

The lipoprotein lipase (*LPL*) gene (OMIM #609708) is located on chromosome 8p21.3 and contains 10 exons. The precursor LPL protein comprises 475 amino acids, while the mature LPL protein (excluding the 27-amino-acid signal peptide) comprises 448 amino acids. LPL is the rate-limiting enzyme for triglyceride (TG) hydrolysis [1, 2]. LPL is mainly synthesized in the parenchymal cells of the heart, skeletal muscle and adipose tissues [3]. It is transported by glycosylphosphatidylinositol-anchored high density lipoprotein-binding protein 1 (GPIHBP1) across endothelial cells to the capillary lumen, where it hydrolyses TG in TG-rich lipoproteins (e.g., chylomicrons and very low–density lipoprotein) [4]. Complete or almost complete loss of LPL function due to biallelic (homozygous or compound heterozygous) *LPL* variants causes autosomal recessive familial chylomicronemia syndrome (FCS; also known as type I hyperlipoproteinemia or LPL deficiency), which is characterized by extremely high plasma TG levels (> 10 mmol/L (880 mg/dL)) [5]. FCS typically develops in infancy or early childhood, manifesting as a failure to thrive, abdominal pain, nausea and vomiting progressing to acute pancreatitis (AP) and various other symptoms (e.g., fatigue, irritability, lipemia retinalis, eruptive xanthomas on trunk, back and gluteal region, and hepatosplenomegaly) [5]. Partial loss of LPL function due to monoallelic (simple heterozygous) *LPL* variants is associated with hypertriglyceridemia (HTG) [6, 7].

Severe HTG is a frequent cause of AP [8]. Indeed, HTG has become the second most common cause of AP in China, accounting for 14–40% of all AP patients [9–11]. HTG-related AP (HTG-AP) is more severe and is associated with poorer outcomes than AP due to other etiologies [9]. Determining the molecular basis of the genetic predisposition to HTG-AP promises to improve our options for both prevention and treatment of the disease. During our routine analysis of genetic risk factors in patients with HTG-AP, including HTG-AP in pregnancy, we identified two novel *LPL* frameshift coding variants (defined here as simple insertion, simple deletion or complex indel variants that (i) occurred entirely within the coding sequence of the *LPL* gene AND (ii) resulted in the net addition or deletion of an odd number of nucleotides at the DNA sequence level). *LPL* frameshift coding variants usually result in the complete functional loss of the affected allele, thereby providing a good model system with which to contrast the complete loss of LPL function (due to biallelic variants) with the partial loss of LPL function (due to monoallelic variants) in human disease. Herein, we describe the identification of two novel *LPL* frameshift variants together with several new insights into the genotype–phenotype relationship obtained through an exploration of the *LPL* frameshift coding variants reported to date.

## Methods

### Ethics statement

This study was approved by the Ethics Committee of Jinling Hostipal, Nanjing, China. Informed consent was obtained from each participant.

### Patient description

Diagnoses of HTG-AP and HTG-AP in pregnancy were made as previously described [12, 13]. Patients #1 and #2 both suffered from HTG-AP during pregnancy.

Patient #1 was a 33-year-old pregnant woman (31^+4^ weeks of gestation). She was admitted to the emergency department due to heavy paroxysmal upper abdominal pain and vomiting. Blood examination revealed chylomicronemia, while the TG level was too high to measure accurately. The serum level of amylase was 1235 U/L, and ultrasound examination noted the presence of peripancreatic fluid collection. On the second day, she received an emergency cesarean section for fetal distress, while 200 mL of chylous abdominal exudate was noted during the operation. After delivery, the patient underwent abdominal computed tomography, revealing typical morphology of AP. On the fifth day, she retained a TG level as high as 23.80 mmol/L (2106.3 mg/dL). She was discharged 17 days later, by which time her fasting TG level had decreased to 4.86 mmol/L (430.11 mg/dL). The patient did not report either a personal or a family history of any disease, with the exception of gestational diabetes mellitus being diagnosed at 28 weeks of gestation.

Patient #2 was a 28-year-old female. She experienced an episode of AP at 38^+4^ weeks of gestation, with a fasting plasma TG level of 35.35 mmol/L (3128.48 mg/dL). She had received symptomatic and organ function support treatments at a local hospital, but her condition did not improve. She was then transferred to our acute pancreatitis center, where she was diagnosed with moderately severe AP with the complication of acute pancreatic necrosis collection. She received comprehensive treatments, including nutritional support and paracentesis drainage. She was discharged 16 days later, by which time her fasting TG level had decreased to 2.59 mmol/L (229.22 mg/dL). The patient did not report any personal or family history of any disease.

Patient #3 was a 43-year-old female. She suffered from sudden and heavy postprandial abdominal pain with nausea and vomiting. She was immediately referred to a local hospital, where a diagnosis of HTG-AP was made (TG level, 33 mmol/L (2920.5 mg/dL)). Twelve days later, she was transferred to our center due to dyspnea and hyperpyrexia and was diagnosed with severe AP. She was discharged 18 days after admission to our hospital with a fasting TG level of 4.94 mmol/L (437.19 mg/dL)). The patient reported a three-year history of HTG. No other personal or family histories of any disease were documented.

### Genetic analysis

Genomic DNA was prepared from peripheral blood cells by means of a Gentra Puregene Blood kit (Qiagen, Dusseldorf, Germany). All exons and flanking intronic regions of five primary HTG-related genes, *LPL, APOA5* (apolipoprotein A5; OMIM #606368)*, APOC2* (apolipoprotein C2; OMIM #608083), *LMF1* (lipase maturation factor 1; OMIM #611761) and *GPIHBP1* (OMIM #612757), were amplified by polymerase chain reaction (PCR) and subsequently Sanger sequenced as previously described [14, 15]. The two novel *LPL* variants reported in this study were confirmed by independent PCR amplification and Sanger sequencing and have been deposited in the ClinVar database (https://www.ncbi.nlm.nih.gov/clinvar/).

### Collation and evaluation of previously reported *LPL* frameshift coding sequence variants

Previously reported *LPL* frameshift coding sequence variants were derived primarily from the Human Gene Mutation Database (HGMD; https://www.hgmd.cf.ac.uk) [16]. This was complemented by a combination of a keyword search (“LPL” or “lipoprotein lipase” plus “frameshift variant”, “frameshift mutation”, “truncation variant”, “truncation mutation”, “termination variant” or “termination mutation”) in PubMed and cross-reference examination. Corresponding original reports were manually evaluated for the following information: zygosity status of the variant, plasma LPL activity of the variant carrier, disease referred for genetic analysis, patient’s age at genetic analysis, and patient’s disease history. Whenever a variant was detected as simple heterozygotes and homozygotes or compound heterozygotes, only the homozygous or compound heterozygous state was considered in this study, the underlying logic being that homozygotes or compound heterozygotes were usually more extensively described in terms of their accompanying clinical genetic data and hence potentially more informative with respect to the genotype–phenotype relationship. Moreover, whenever an included variant was reported in ≥ 2 publications, only the first publication was cited. Furthermore, whenever a variant of interest was coinherited with an *LPL* missense variant in *trans*, the LPL activity of the missense variant determined by cell transfection experiments was collated by means of a literature search. Variant collation and literature review were frozen on 15 March 2023.

### SpliceAI prediction

SpliceAI, a 32-layer deep neural network [17] and currently the most accurate tool for predicting splicing variants, was employed to predict the impact of *LPL* frameshift coding variants and a common *LPL* intronic variant (i.e., c.1428-11C > T) on mRNA splicing. The prediction was performed at https://spliceailookup.broadinstitute.org/ (last accessed on July 26, 2023) using the following parameters: Genome version, hg38; Score type, masked; Max distance, 10000. The Δ score ranges from 0 to 1; the higher the score, the more likely the variant is to affect splicing. A variant with a Δ score of ≤ 0.20 was considered unlikely to affect splicing.

### Reference sequences and variant nomenclature

NM_000237.3 and NC_000008.11 were used as reference *LPL* mRNA and DNA sequences, respectively. All variants were named in accordance with Human Genome Variation Society (HGVS) recommendations (http://varnomen.hgvs.org/) [18], with the A of the translation initiation ATG codon of the *LPL* gene being counted as nucleotide + 1. All variant names at the coding DNA level were verified/normalized at the Leiden University Medical Center (LUMC) Mutalyzer 3 website (https://mutalyzer.nl/) [19]. Variant names at the protein level also followed LUMC Mutalyzer 3 except for one variant (i.e., c.767_768insTAAATATT) that was experimentally determined to result in a transcript with an in-frame deletion [20]. It is also worth mentioning that variants reported in the early literature were described in the context of the 448-amino-acid mature LPL protein (N.B. The first nucleotide of the mature LPL protein-coding sequence corresponds to position c.82 of NM_000237.3; c.1_81 of NM_000237.3 encodes the 27-amino-acid signal peptide of the LPL preprotein).

## Results

### Identification of two novel *LPL* frameshift coding sequence variants

**Fig. 1:**
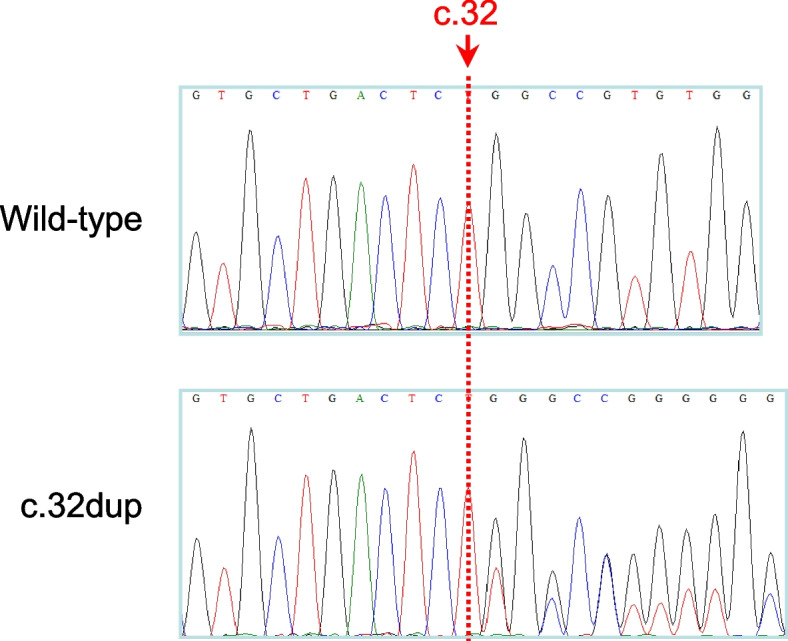
Sanger sequencing electropherogram showing the heterozygous single-nucleotide duplication c.32dup in exon 1 of the *LPL* gene. The variant has been submitted to the ClinVar database

We first focused on rare (allele frequency of < 1% in all gnomAD (Genome Aggregation Database; https://gnomad.broadinstitute.org/) populations) missense, frameshift, nonsense or splice site variants in the five primary HTG-related genes, *LPL*, *APOA5*, *APOC2*, *LMF1* and *GPIHBP1*. In patients #1 and #2, we identified the same heterozygous single-nucleotide duplication in exon 1 of the *LPL* gene, c.32dup (Fig. 1). This variant was deemed to cause a complete functional loss of the affected allele by virtue of its frameshift nature; at the protein level, it was termed p.(Ala12Glyfs*29). The two patients were not known to be related. However, it remains possible that the disease alleles were identical by descent.

**Fig. 2:**
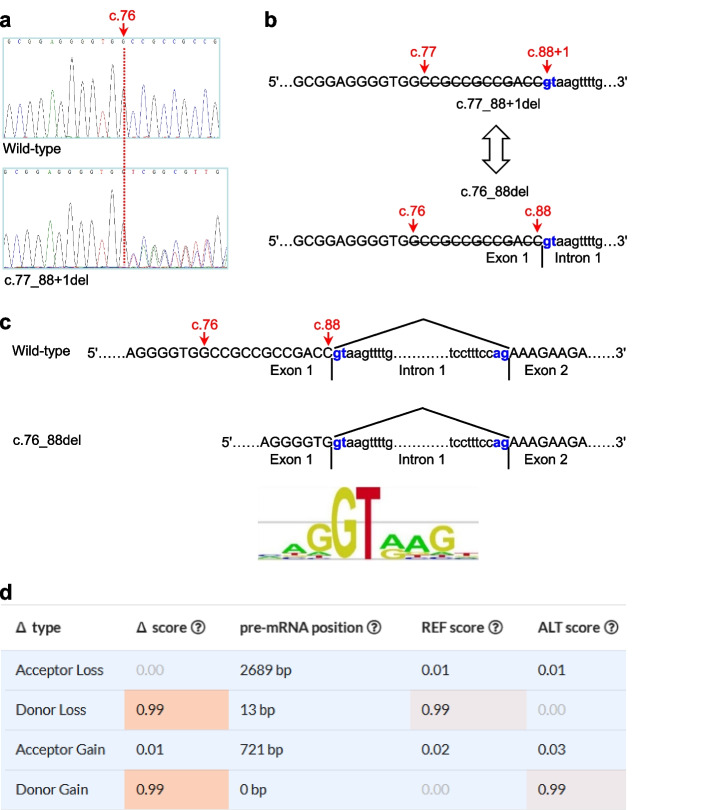
Identification of a heterozygous 13-bp deletion in the *LPL* gene and in silico prediction of its impact on splicing. **a** Sanger sequencing electropherogram showing the 13-bp deletion c.77_88 + 1del (nomenclature in accordance with Human Genome Variation Society (HGVS) recommendations). The variant has been submitted to the ClinVar database. **b** Illustration showing that c.77_88 + 1del can be alternatively described as c.76_88del. The exon 1 sequence is shown in the upper case, whereas the intron 1 sequence is shown in the lower case. The canonical 5′ splice site GT dinucleotide is highlighted in bold and blue. The start and end positions of the 13-bp deletion in the two alternative nomenclature versions are indicated by red arrows. **c** Presumed splicing of the c.76_88del *LPL* pre-mRNA. Normal splicing of the wild-type *LPL* pre-mRNA is shown for comparison. The DNA sequence was used here instead of the RNA sequence for illustrative purposes. The obligate dinucleotides from the donor and acceptor splice sites, gt and ag, are highlighted in bold and blue. It should be noted that the sequence spanning the junction of the c.76_88del allele conformed to the 5′ splice site consensus sequence. The position weight matrix of the 9‐bp 5′ splice site signal sequence was taken from [22], an Open Access article distributed under the terms of the Creative Commons Attribution Noncommercial License. **d** SpliceAI predicted impact of c.76_88del on splicing. See text for data interpretation

In patient #3, we identified a heterozygous 13-bp deletion in the *LPL* gene (Fig. 2a). The 13-bp deletion was termed c.77_88 + 1del in accordance with HGVS recommendations, but it could alternatively be described as c.76_88del (Fig. 2b), thereby fulfilling the definition of a frameshift coding sequence variant (N.B. The HGVS recommends that “for deletions, duplications and insertions, the most 3’ position possible is arbitrarily assigned to have been changed”). For ease of discussion, this latter description was used here to explore the functional consequences of the 13-bp deletion. As illustrated in Fig. 2c, the sequence spanning the junction of c.76_88del appeared to concur with the 5’ splice site consensus sequence (see [21] and references therein). This suggested that the mutant pre-mRNA would not differ from the wild-type pre-mRNA in terms of intron 1 splicing. As such, the 13-bp deletion would also cause a complete functional loss of the affected allele by virtue of its frameshift nature; at the protein level, it was termed p.(Ala26Lysfs*13).

Neither c.32dup nor c.77_88 + 1del has previously been reported in the literature. Neither of them is present in gnomAD or ClinVar (https://www.ncbi.nlm.nih.gov/clinvar) (as of 25 May 2023). We did not identify any other rare missense, frameshift, nonsense or splice site variants in the five primary HTG-related genes, *LPL, APOA5, APOC2, LMF1* and *GPIHBP1,* in any of the three patients.

We did not identify any rare synonymous or intronic variants in the five primary HTG-related genes in any of the three patients. Variants with an allele frequency of ≥ 1% found in the three patients are listed in Supplementary Table 1. With the exception of *LPL* c.1428-11C > T (N.B. this variant was predicted here, by means of SpliceAI, to have no effect on splicing), all these variants were annotated as “benign” in terms of their likely clinical significance by gnomAD. This notwithstanding, some of these variants may have functional consequences and may therefore predispose to or protect against HTG or HTG-related disorders. However, such situations would not affect the pathological relevance of the two novel *LPL* frameshift coding variants and hence would not alter the main conclusions of this study. Consequently, those variants with an allele frequency of ≥ 1% will not be discussed further in the manuscript.

### Exploration of the *LPL* frameshift coding variants reported thus far

We collated 53 previously reported *LPL* frameshift coding variants (Table 1) through a combination of data acquired from HGMD, PubMed keyword search and cross-reference examination. Here, it should be emphasized that c.247_249 + 1del was included as a frameshift coding variant since it could be alternatively described as c.246_249del (i.e., deletion of the last four nucleotides of exon 2; Supplementary Fig. 1).

**Table 1:** Summary of the key clinical genetic data with respect to the 55 *LPL* frameshift coding variants reported to date

Variant			Zygosity	The other variant in case of compound heterozygosity (functional effect)^b^	Plasma LPL activity (% of normal)	Disease referred for genetic analysis	Patient’s age at genetic analysis	Patient’s disease history
**HGVS nomenclature** **(NM_000237.3)**	**Original description**^**a**^	**Reference**						
c.10_11insTTCG p.(Ala117Serfs*61)		[23]	Heterozygote		NI^c^	Severe HTG	NI	NI
c.32dup p.(Ala12Glyfs*29)		This study	Heterozygote		No data	HTG-AP in pregnancy	#1: 33 y (31^+4^ weeks of gestation) #2: 28y (38^+4^ weeks of gestation)	#1: Gestational diabetes mellitus at 28 weeks of gestation #2: negative
c.46_47del p.(Gln16Glufs*24)	Q-12E > 11X^d^	[24]	Heterozygote		NI	Severe HTG	NI	NI
c.77_88 + 1del p.(Ala26Lysfs*13)		This study	Heterozygote		No data	HTG-AP	43 y	Three years of HTG
c.94_98del p.(Arg32Phefs*7)		[25]	Heterozygote		NI	HTG	NI	NI
c.128dup p.(Arg44Lysfs*4)		[26]	Compound heterozygote	p.Asn318Ser (experimentally determined to have 60% wild-type LPL activity by another study [27])	NI	Eruptive cutaneous xanthomata and elevated plasma TG concentration	29 y	Negative
c.133_143del p.(Thr45Hisfs*3)	11 bp deletion in exon 2	[28]	Compound heterozygote	p.Gly215Glu^e^ (experimentally determined to have a near complete functional loss of LPL by two studies [29, 30])	< 1%	Familial chylomicronemia	10 y	Marked HTG at birth
c.133dup p.(Thr45Asnfs*3)	c.134insA	[31]	Heterozygote		NI	Moderate HTG	41 y	NI
c.183dup p.(Glu62Argfs*28)	Insertion of an "A" at nucleotide 183 (codon Glu35)	[32]	Homozygote		0	Familial chylomicronemia	5.5 y	Chylomicronemia at 1 month of age after an episode of AP
c.247_249 + 1del	4-bp ACGG deletion at the 3' boundary of exon 2	[33]	Unknown^f^		Unknown	LPL deficiency	Unknown	Unknown
c.287_288del p.(Val96Glyfs*51)	Deletion of nucleotides G^286^ and T^287^ in exon 3	[34]	Compound heterozygote	c.440_443del^g^ p.(Asn147Thrfs*24)	0	Chylomicronemia	17 y	Manifested with failure to thrive and abdominal pain at age 3 weeks
c.289_294delinsTTTGCCAAAA p.(Ala97Phefs*52)	Wrongly described as c.289_299delGCCGCCinsTTTGCCAAAA	[35]	Homozygote		NI	Very severe HTG and cerebral dysmorphism	2 m	NI
c.290_293delinsGG p.(Ala97Glyfs*50)	Deletion of four nucleotides (∆CCGC) and an insertion of two nucleotides (∇GG) at position 290 in exon 3	[36]	Compound heterozygote	p.Leu313Pro^h^ (experimentally shown to have < 1% of wild-type LPL activity [36])	6.6%	Familial Chylomicronemia	67 y	Eruptive xanthomata and chylomicronemia were noted at the age of 53, during an episode of acute pancreatitis
c.312del p.(Asp105Thrfs*67)		[37]	Homozygote		0	FCS	3 y	NI
c.334dup p.(Asp112Glyfs*36)		[38]	Compound heterozygote	p.Arg102Thr (both p.Arg102Thr and p.(Asp112Glyfs*36) were experimentally shown to cause a complete functional loss of LPL [38])	NI	Unexplained fever (“Milky serum” was noticed during laboratory examination)	1 m	
c.338_339insAGAGTACCATTCGATAC p.(Trp113*)		[23]	Heterozygote		NI	Severe HTG	NI	NI
c.348_349insAGTACCATTCGACAGTC p.(Ala117Serfs*61)		[23]	Heterozygote		NI	Severe HTG	NI	NI
c.373dup p.(Ala125Glyfs*23)	c.373_374insG	[39]	Compound heterozygote	p.His273Arg (experimentally determined to have 2% of wild-type LPL activity [39])	NI	Severe HTG	3 y	NI
c.377_378insAGAGTACCATT p.(Tyr127Glufs*49)		[23]	Heterozygote		NI	Severe HTG	NI	NI
c.384delinsTGGGCT p.(Lys129Glyfs*45)	A six base-pair insertion at the site of a single base deletion, and that the net insertion of five base-pairs at amino acid positions 102 to 103 causes a shift in the reading frame	[40]	Unknown		Unknown	LPL deficiency	Unknown	Unknown
c.386_390del p.(Lys129Serfs*17)		[31]	Heterozygote		NI	Severe HTG	46 y	NI
c.431_432AG[3] p.(Glu145Argfs*4)	c.429_430insGAGA	[23]	Heterozygote		NI	Severe HTG	NI	NI
c.438del p.(Phe146Leufs*26)		[41]	NI		NI	HTG	NI	NI
c.440_443del p.(Asn147Thrfs*24)	A 4 bp deletion (ACTA) in exon 4	[34]	Compound heterozygote	c.287_288del (described earlier in the Table)	0	Chylomicronemia	17 y	Manifested with failure to thrive and abdominal pain at age 3 weeks
c.483delA p.(Ala162Profs∗10)		[42]	Heterozygote		NI	Glycogen storage disease type-Ib (severe HTG discovered during laboratory tests)	5 m	Hypoglycemia, hyperlactic acidosis, and sepsis in the neonatal period
c.501_502insGAGAGTACCATTCGAGA p.(Ala168Glufs*10)		[23]	Heterozygote		NI	Severe HTG	NI	NI
c.596del p.(Ser199Phefs*8)	Deletion of the second nucleotide of codon 172; p.S172fsX179	[43]	Heterozygote		30%	Recurrent hypertriglyceridemic pancreatitis	50 y	Found to be hypertriglyceridemic for 30 years and suffered from four episodes of acute pancreatitis between the ages of 46 y and 49 y
c.599del p.(Pro200Leufs*7)		[44]	Heterozygote		NI	HTG	NI	NI
c.624del p.(Leu209Tyrfs*43)		[45]	Heterozygote		NI	NI	NI	NI
c.651del p.(Gly218Valfs*34)		[35]	Homozygote		NI	Severe HTG	4 y	Pancreatitis at the age 2 y
c.708del p.(Gly237Valfs*15)	Deletion of the third nucleotide of the codon for Gly209, resulting in termination after 223 residues	[46]	NI		NI	Severe HTG	NI	NI
c.742del p.(Ala248Leufs*4)	Deletion of a C at base 916	[47]	Homozygote		0 (in both diseased siblings)	Type I hyperlipoproteinemia	NI	Both patients had recurrent episodes of abdominal pain and pancreatitis (age of first disease onset not described)
c.765_766del p.(Gly256Thrfs*26)	c.765_766delAG	[48]	Homozygote		0 (in two patients from a same family)	Type I hyperlipoproteinemia	#1: 19 y #2: 32 y	#1: NI #2: NI
c.767_768insTAAATATT p.(Gly256_Gly258del)		[20]	Homozygote		NI	HTG-AP	25 y	Central abdominal pain on two occasions in the previous two years; without any other syndromes or diseases
c.769_770insCA p.(Leu257Profs*8)		[23]	Heterozygote		NI	Severe HTG	NI	NI
c.835_836del p.(Leu279Valfs*3)		[35]	Homozygote		NI	Severe HTG (two patients from a same family)	#1: 37 y #2: 29 y	#1: NI #2: Recurrent pancreatitis starting from the age 1 y
c.840del p.(Asn281Metfs*23)		[35]	Compound heterozygote	Gross deletion of the *LPL* gene		Familial chylomicronemia	1 m	Had severe HTG, hepatomegaly, lipemia retinalis and eruptive xanthomas
c.899_921dup p.(Asn308Glyfs*4)		[49]	Homozygote		NI	Severe HTG	NI	HTG since childhood
c.901del p.(Leu301Serfs*3)		[49]	Heterozygote		23%	Severe HTG	39 y	NI
c.953del p.(Asn318Ilefs*13)	One nucleotide deletion of A coding Asn 291	[50]	Compound heterozygote	p.Ile221Thr^i^ (almost complete loss of LPL activity [51, 52])	7.5%	Severe HTG	33 y	Suffered from AP several times after drinking alcohol. He did not have diabetes, renal disease, liver disease or hormonal disease. No corneal opacification, xanthomatosis or hepatosplenomegaly were noted
c.1008del p.(Met336Ilefs*10)		[45]	Heterozygote		NI	NI	NI	NI
c.1010_1011insATTCGAGAGC p.(Tyr338Phefs*19)	c.1009_1010insCATTCGAGAG	[23]	Heterozygote		NI	Severe HTG	NI	NI
c.1016_1017insC p.(Lys339Asnfs*15)	AAA → AACA in exon 6	[53]	Heterozygote		NI	HTG (reported in a family; all six variant carriers had HTG while all noncarriers had normal TG levels)	NI	NI
c.1044_1050del p.(His348Glnfs*43)		[49]	Heterozygote		NI	Severe HTG	52 y	NI
c.1081_1082insAGTA p.(Ala361Glufs*4)		[23]	Heterozygote		NI	Severe HTG	NI	NI
c.1107_1108insATTCGAAGAGCGC p.(Val370Ilefs*9)	c.1106_1107insCATTCGAAGAGCG	[23]	Heterozygote		NI	Severe HTG	NI	NI
c.1115dup p.(Ser373Glufs*2)		[23]	Heterozygote		NI	Severe HTG	NI	NI
c.1119_1120insACCATTC p.(Glu374Thrfs*11)		[23]	Heterozygote		NI	Severe HTG	NI	NI
c.1121_1122insAGAGCGC p.(Asn375Glufs*10)		[23]	Heterozygote		NI	Severe HTG	NI	NI
c.1138_1139del p.(Leu380Alafs*2)	Deletion CT_1312–1313_ that covers the last two bases of exon 7	[54]	Homozygote		0 (in both the proband and his sister)	Severe HTG	Proband: 17.1 y Sister: 8.9 y	Proband: recurrent episodes of abdominal pain and unexplained diarrhea; acute pancreatitis at age 5 years Sister: severe HTG
c.1160_1161insT p.(Lys387Asnfs*26)		[55]	Homozygote		NI	LPL deficiency	NI	NI
c.1163_1164insA p.(Tyr389Leufs*24)	ACC → ACAC in exon 8	[53]	Heterozygote		NI	HTG (variant carriers had significantly higher TG levels than noncarriers based upon clinical data from 15 variant carriers and 17 noncarriers	NI	NI
c.1303_1304del p.(Ala435Argfs*12)		[23]	Heterozygote		NI	Severe HTG	NI	NI
c.1306_1307insAGTACCATTC p.(Gly436Glufs*15)		[23]	Heterozygote		NI	Severe HTG	NI	NI
c.1373del p.(Ala458Aspfs*6)		[56]	Homozygote		NI	LPL deficiency	2 m	Patient exhibited lipemic plasma, lipemia retinalis, hepatomegaly, splenomegaly, and failure to thrive

*Abbreviations*: *AP* acute pancreatitis, *HTG* hypertriglyceridemia *LPL* lipoprotein lipase, *M* months, *NI* not informative, *TG* triglyceride, *Y* years

^a^For variants that were either not described at the coding DNA reference sequence level or described at the coding DNA reference sequence level but the nomenclature did not follow the current Human Genome Variation Society (HGVS) recommendations

^b^Functional effect refers to the experimentally determined LPL activity (in the medium of transfected cells) of the *trans*-inherited *LPL* missense variant relative to that of wild-type LPL

^c^Not informative in the original publication

^d^Described only at the protein level in the first report [24]. The change at the coding DNA sequence level was obtained from subsequent citing publications [57, 58]

^e^Described as Gly188Glu in the original report [28]

^f^Full text of the original report could not be accessed

^g^Described as a 4 bp deletion (ACTA) in exon 4 in the original report [34]

^h^Described as Leu286Pro in the original report [36]

^i^Described as LPL p.Ile194Thr in the original report [50]

**Fig. 3:**
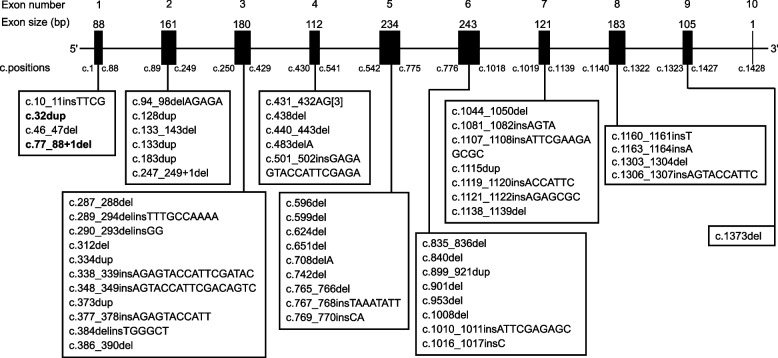
Exon locations of the 55 *LPL* frameshift coding variants reported to date. The two novel variants reported in the present study are highlighted in bold. c.77_88 + 1del and c.247_249 + 1del were included because they can be alternatively described as c.76_88del and c.246_249del, respectively. Bars indicate exons. Note that for exons 1 and 10, only the coding sequences are shown. c.positions, start and end coding positions of each exon in accordance with NM_000237.3

The 53 previously reported *LPL* variants, together with the 2 newly reported events, were spatially positioned according to their affected exons (Fig. 3). Allowing for variable exon size, a paucity of frameshift coding variants was evident in exon 9 compared to the preceding exons. However, this may represent a chance finding owing to the small sample size.

We reviewed the corresponding original reports with respect to variant zygosity and the patient’s clinical and laboratory characteristics for each variant (Table 1). Two variants (c.247_249 + 1del [33] and c.384delinsTGGGCT [40]) were “unknown” for zygosity status since we were unable to access the full texts of the original publications. The zygosity status of two other variants (c.438del [41] and c.708del [46]) was “uninformative” due to the lack of relevant information in the original publications. These four variants were therefore excluded from further discussion in terms of genotype–phenotype relationships. Of the 51 variants informative for zygosity, 30 were detected as simple heterozygotes, 12 were detected as homozygotes, and 9 were detected as compound heterozygotes.

#### Genotype–phenotype correlations in individuals with simple heterozygous variants

Simple heterozygous *LPL* frameshift coding variants were almost invariably reported in patients with severe HTG and HTG-related diseases such as HTG-AP or HTG-AP in pregnancy. Ages at genetic analysis in these patients, whenever informative, were almost invariably > 20 years. c.483delA represents a notable exception: it was identified in a 5-month-old girl with glycogen storage disease type Ib, and the carrier was found to have severe HTG during laboratory tests in relation to her primary disease [42]. It is possible that glycogen storage disease type Ib may have precipitated the early-onset occurrence of severe HTG in this particular case. Alternatively, this patient may harbor additional as yet undiscovered pathogenic variant(s) in the *LPL* gene or other HTG-related genes.

#### Genotype–phenotype correlations in individuals with homozygous variants

Of the 12 homozygous variants identified, 11 can be assigned as causative for typical FCS or LPL deficiency based upon disease phenotype, age of disease onset and/or in vivo LPL activity (Table 1). However, the remaining variant, c.767_768insTAAATATT, was identified in a 25-year-old woman with HTG-AP [20]. The patient reported abdominal pain on two occasions in the previous two years but otherwise had no symptoms of FCS. This rather mild phenotype (by reference to typical FCS) was accounted for by the fact that c.767_768insTAAATATT turned out to be an in-frame variant at the mRNA level, as revealed by RT‒PCR analysis of RNA prepared from the patient’s peripheral blood cells. Specifically, c.767_768insTAAATATT abolished the physiological GT donor site of intron 5 while creating a new splice donor site spanning the 5’ insertion junction, resulting in the generation of a transcript with a 9-bp in-frame deletion (precisely, the deletion of the last 9 nucleotides of exon 5 of the wild-type *LPL* gene); the mutant transcript would encode a protein with an in-frame deletion of 3 amino acids (i.e., Gly256, Leu257 and Gly258); the mutant protein lacking the three amino acids has been considered to be partially functional [20]. Thanks to the availability of the RT‒PCR analysis data, c.767_768insTAAATATT could confidently be termed p.Gly256_Gly258del at the protein level. Otherwise, it would have been termed p.(Leu257Lysfs*10) in accordance with LUMC Mutalyzer 3.

In summary, of the 12 frameshift coding variants identified in the homozygous state, one (c.767_768insTAAATATT) was conclusively demonstrated to retain residual LPL function based upon both a combination of clinical information and functional analysis data.

#### Genotype–phenotype correlations in individuals with compound heterozygous variants

Of the 9 variants identified as components of compound heterozygotes, c.287_288del and c.440_443del were identified in the same subject with FCS, who manifested with failure to thrive and abdominal pain at 3 weeks of age [34]. A third variant, c.840del, which was coinherited with a gross deletion of the *LPL* gene, was identified in a one-month-old subject with typical FCS [35]. The typical FCS associated with these three frameshift coding variants implied that all three of these variants should result in the complete or almost complete loss of LPL function. For the remaining 6 variants, the *LPL* variants inherited in *trans* were invariably missense variants. These six compound heterozygous variants will now be addressed individually.

c.128dup and p.Asn318Ser: these two variants were found in a 29-year-old patient with eruptive cutaneous xanthomata and an elevated plasma TG concentration [26]. This late onset of disease could in principle be accounted for by the residual function of p.Asn318Ser, which had been experimentally determined to exhibit 60% wild-type activity [27].

c.133_143del and p.Gly215Glu: their carrier had barely detectable LPL activity (< 1% of control levels), exhibited marked HTG at birth and was diagnosed as having FCS in childhood [28]. Consistent with this picture, p.Gly215Glu was experimentally determined to lead to a near complete functional loss of LPL [29, 30].

c.290_293delinsGG and p.Leu313Pro: the male carrier was described to have FCS [36]. However, he was subjected to genetic analysis at the age of 67, having previously reported eruptive xanthomata and chylomicronemia during an episode of AP at the age of 53. Moreover, the patient’s plasma LPL activity was found to be 6.6% of that of controls. These pieces of clinical and biological evidence converged with the conclusion that the patient had a milder phenotype than typical FCS. However, this comparatively mild phenotype would be at odds with p.Leu313Pro’s experimentally determined LPL activity (i.e., < 1% of wild-type [36]). To account for this discrepancy, we speculate either that c.290_293delinsGG may retain some residual LPL function or that the in vitro determined functional loss of the p.Leu313Pro missense variant may not reflect the variant’s in vivo properties.

c.334dup and p.Arg102Thr: these variants were found in a one-month-old baby exhibiting severe HTG [38]. Consistent with this phenotype, both variants were experimentally shown to result in the complete functional loss of the affected *LPL* allele [38].

c.373dup and p.His273Arg: these variants were identified in a 3-year-old child with severe HTG [39]. Consistent with this early onset of disease, p.His273Arg was experimentally determined to exhibit 2% wild-type activity [39].

c.953del and p.Ile221Thr: the male carrier of these variants exhibited a relatively mild clinical phenotype compared to typical FCS [50]. First, he was genetically tested for severe HTG at the age of 33, having not previously reported any other symptoms of FCS. Second, his plasma LPL activity was 7.5% of that of controls. However, p.Ile221Thr was experimentally shown to be associated with an almost complete loss of LPL activity by two different studies [51, 52], a finding supported by two clinical findings. First, p.Ile221Thr homozygosity was found in a patient with LPL deficiency (patient presented at the age of 4 years with a history of hepatosplenomegaly and abdominal pain; plasma lipid analysis revealed chylomicronemia with TG levels in excess of 30 mmol/L; plasma LPL activity in this patient was undetectable) [51]. Second, p.Ile221Thr and another missense variant, p.Arg270His (experimentally shown to be associated with a complete functional loss variant), were identified in a typical FCS patient who exhibited HTG at birth, had recurrent episodes of abdominal pain and pancreatitis and was noted to have eruptive xanthomas, lipemia retinalis, and splenomegaly throughout childhood [52]. The in vitro functional analysis data and clinical information firmly established that p.Ile221Thr caused complete functional loss of the affected allele. Consequently, the mild phenotype of the c.953del and p.Ile221Thr compound heterozygote should be conferred by the c.953del allele, which might retain some residual LPL function.

In short, of the 9 frameshift coding variants identified in the compound heterozygous state, one (c.953del) was reasoned to retain some residual LPL function.

### Exploring the possibility that some *LPL* frameshift coding variants may generate “in-frame” transcripts

As mentioned earlier, c.767_768insTAAATATT homozygosity was associated with a mild phenotype. Consistent with this, c.767_768insTAAATATT has been experimentally shown to affect splicing, thereby generating a transcript with a 9-bp in-frame deletion [20]. This prompted us to explore whether some other *LPL* frameshift coding variants could have a similar effect. To this end, we predicted the potential impact of all 55 *LPL* variants by means of SpliceAI (Supplementary Fig. 2). Seven variants were predicted to have at least one Δ score of > 0.20. These seven variants will be addressed first.

#### c.767_768insTAAATATT

This variant was predicted both to disrupt the physiological splice donor site of intron 5 (Δ score of donor loss, 0.85; premRNA position, 8 bp) and to create a new splice donor site spanning the 5’ insertion junction (Δ score of donor gain, 1.0; premRNA position, -1 bp) (Supplementary Fig. 2). These predictions are entirely consistent with the findings from the RT‒PCR analysis of patient-derived blood cells [20].

#### Three variants with high Δ scores but with no impact on splicing

Three variants (i.e., c.76_88 (HGVS name, c.77_88 + 1del), c.246_249del (HGVS name, c.247_249 + 1del) and c.1138_1139del) were similar in two respects. First, all variants served to delete the last nucleotides of the exons they affected. Second, they were predicted to have the highest Δ scores in terms of both splicing types (i.e., donor loss and donor gain) and values (0.99 to 1.00) (Supplementary Fig. 2). Evaluations of the corresponding donor gain and loss positions, however, revealed that their respective wild-type and mutant pre-mRNA sequences did not differ in terms of their consequences for splicing. Taking c.76_88 as an example (Fig. 2d), a Δ score of 0.99 for donor loss and a Δ score of 0.99 for donor gain suggested that the deletion allele had the same potential for splicing as the wild-type allele. The donor loss at the pre-mRNA position 13 bp in the context of the wild-type allele refers to c.88, whereas the donor gain at pre-mRNA position 0 bp in the context of the deletion allele refers to c.75. This indicates that the wild-type and c.76_88 pre-mRNAs were identical in terms of intron 1 splicing, as depicted in Fig. 2c. The mutant c.76_88del mRNA sequence (lacking the last 13 nucleotides of exon 1 compared to the wild-type transcript) would thus be predicted to encode a significantly truncated and nonfunctional protein, p.(Ala26Lysfs*14).

In short, the three variants did not affect splicing despite their very high Δ scores, thereby excluding the possibility of generating aberrantly spliced but “in-frame” transcripts. In other words, all three variants are expected to result in the complete functional loss of their affected alleles. Here, it should be noted that one of these variants was informative with respect to in vivo LPL activity; consistent with our predictions, both c.1138_1139del homozygotes exhibited zero plasma LPL activity [54].

#### One variant potentially affects splicing and leads to the generation of some “in-frame” transcripts

**Fig. 4:**
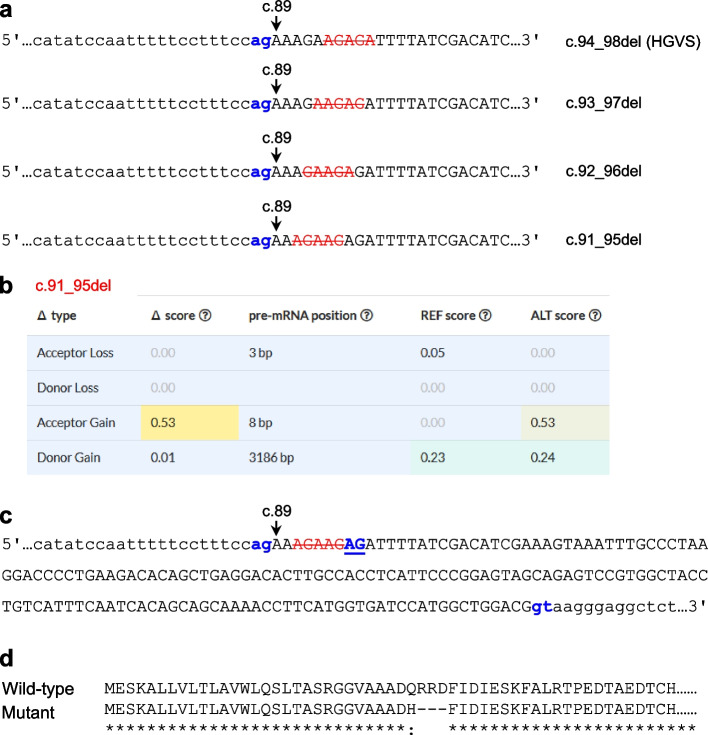
Possible residual function of the *LPL* c.94_98del variant. **a** Illustration showing that c.94_98del (nomenclature in accordance with Human Genome Variation Society (HGVS) recommendations) can be alternatively described as c.93_97del, c.92_96del or c.91_95del. The exon 1 sequence is shown in uppercase letters, whereas the intron 1 sequence is shown in lowercase letters. The canonical 3′ splice site ag dinucleotide is highlighted in bold and blue. Deleted nucleotides in the different nomenclature versions are barred and red. **b** SpliceAI-predicted results for c.91_95del. **c** *LPL* exon 2 and flanking intronic sequences. The exon 1 sequence is shown in uppercase letters, whereas intronic sequences are shown in lowercase letters. The physiological obligate acceptor and donor dinucleotides (ag and gt) are highlighted in blue. The c.91_95del variant (red and barred) was predicted by SpliceAI to activate a downstream cryptic splice acceptor site (highlighted in blue and underlined). The use of this cryptic splice acceptor site would result in a transcript lacking the first 9 bp of exon 2. **d** Alignment of the mutant and wild-type LPL preproteins

C.94_98del can be alternatively described as c.93_97del, c.92_96del or c.91_95del (Fig. 4a). Using c.91_95del for prediction, the variant had an acceptor gain Δ score of 0.53 (at position 8 bp) (Fig. 4b), indicating the activation of a cryptic acceptor site immediately after the deleted nucleotides (Fig. 4c). The aberrantly spliced transcript would be predicted to lack the first 9 nucleotides of exon 2 (Fig. 4c), thereby encoding a protein with a missense variant (p.Gln30His) followed by the deletion of three amino acids (p.Arg31_Asp33del) (Fig. 4d). Given that these changes are located within the amino terminus of the mature LPL protein (starting at p.Ala28), it is highly likely that the mutant protein would retain some residual function. Unfortunately, this postulate cannot be confirmed (or refuted) by available clinical genetics data because c.94_98del was reported in a subject with HTG in the heterozygous state [25]. HTG, unlike autosomal recessive FCS, is a complex and quantitative trait. Finally, it should be noted that c.94_98del was not predicted by SpliceAI to disrupt any physiological splice donor or acceptor sites (Fig. 4b). Consequently, c.94_98del may generate a mixture of “normally spliced but frameshift transcripts” and “aberrantly spliced but in-frame transcripts”.

#### Two variants potentially affecting splicing but that do not generate “in-frame” transcripts

C.899_921dup had a donor loss score of 0.25 and a donor gain score of 0.41 (Supplementary Fig. 3). This suggested that the variant allele might generate an aberrantly spliced transcript in addition to the “normally spliced but frameshift transcript”. However, this aberrantly spliced transcript is unlikely to encode a protein product with any LPL function due to the splicing out of the last 149 (not divisible by 3) nucleotides of exon 6 (Supplementary Fig. 3).

c.1160_1161insT had an acceptor gain score of 0.43 (Supplementary Fig. 4). The aberrantly spliced transcript is also unlikely to encode a protein product with any LPL function due to its splicing out of the first 47 (not divisible by 3) nucleotides of exon 8 (Supplementary Fig. 4).

#### c.953del is among the variants that were not predicted to affect splicing

None of the remaining 48 variants were predicted to have a Δ score of > 0.20 (Supplementary Fig. 2). Interestingly, c.953del, which was previously thought to retain some residual LPL function, was among these variants. c.953del had very low donor loss and gain scores (0.07 and 0.08, respectively; Supplementary Fig. 2).

## Discussion

*LPL* is one of the most extensively studied human disease genes. Hundreds of loss-of-function *LPL* variants resulting from the study of FCS, HTG or HTG-related diseases have been reported in the literature in different combinations and permutations [16]. Herein, subsequent to the identification of two novel *LPL* frameshift coding variants in Chinese patients with HTG-AP or HTG-AP in pregnancy, we embarked on an exploration of the genotype–phenotype relationship in relation to the *LPL* frameshift coding variants reported to date. This analysis generated some interesting findings. First, the study of biallelic variants involving *LPL* frameshift coding variants in cases with typical FCS or milder forms of the disease generated important insights into the clinical importance of the residual function of LPL. Based upon current data on genotype–phenotype correlations, we conclude that 6–7% residual LPL function could significantly delay the disease onset age of FCS as well as reduce the occurrence rate of FCS-associated syndromes. This is comparable to the situation evident with autosomal recessive cystic fibrosis, where 5% normal *CFTR* gene expression is sufficient to prevent the pulmonary manifestations of the disease [59]. Second, whereas a large majority of *LPL* frameshift coding variants completely disrupt gene function through their "frameshift" nature, a small fraction of these variants may act wholly or partly as "in-frame" variants, leading to the generation of protein products with some LPL function. Third, SpliceAI has been widely used to predict the potential effects of different types of variants in many disease genes. For example, we have recently employed it to aid the classification of pancreatitis-associated *PRSS1* [60] and *PNLIP* [61]variants. Herein, SpliceAI perfectly predicted the splicing effect of c.767_768insTAAATATT and suggested that c.94_98del may be capable of generating some “in-frame” transcripts. In contrast, c.953del, which was reasoned by us to retain residual function, was not predicted by SpliceAI to be capable of generating “in-frame” transcripts. These latter two variants would be interesting candidates for in vitro functional analysis.

The frameshift coding sequence variants studied here were defined solely on the basis of their DNA sequence change (see Introduction). Given that this type of variant is not limited to *LPL*, insights generated from this study could have implications for the genotype–phenotype relationship of frameshift coding sequence variants in other disease genes. Herein, it is worth noting that other types of variants, such as missense and synonymous variants, may also lead to "frameshift" or "in-frame" changes at the RNA level by affecting pre-mRNA splicing, but this lies out within the scope of the current study.

### Study strengths and limitations

The strengths of our study were that (i) we reported two novel variants and (ii) we performed the first comprehensive exploration of genotype–phenotype relationships for the *LPL* frameshift coding variants reported thus far. One limitation of our study was the relatively small sample size.

## Conclusions

In this study, we reported 2 novel *LPL* frameshift coding variants, followed by an exploration of genotype–phenotype relationships for the *LPL* frameshift coding variants reported to date. Careful evaluation of the 55 *LPL* frameshift coding variants with respect to their clinical and genetic data generated several novel insights, especially in relation to the importance of residual LPL function in modifying the age of disease onset and subsequent clinical expression in the context of autosomal recessive FCS.

### Supplementary Information

**Additional file 1: Supplementary Table 1**. Variants with an allele frequency of ≥1% found in the three patients. **Supplementary Figure 1**. Illustration showing that c.247_249+1del (in accordance with Human Genome Variation Society (HGVS) recommendations) may be alternatively described as c.246_249del. Exon 2 sequence is shown in upper case letters whereas intron 2 sequence is shown in lower case. The canonical 5’ splice site gt dinucleotide is highlighted in bold and blue. The deleted four nucleotides in each nomenclature version are barred. **Supplementary Figure 2**. SpliceAI predictions for the 55 *LPL* frameshift coding variants reported to date. **Supplementary Figure 3**. SpliceAI-predicted impact of the *LPL* c.899_921dup variant on splicing. (a) SpliceAI predictions. (b) Explanation of the predicted results in the context of the wild-type *LPL* exon 6 and flanking intronic sequences. Exon 6 sequence is shown in upper case letters whereas intronic sequence is shown in lower case. The physiological obligate acceptor and donor dinucleotides (ag and gt) are highlighted in blue. The 23-bp duplicated sequence is highlighted in red. The *LPL* c.899_921dup variant was predicted to activate an upstream cryptic donor splice site (highlighted in blue and underlined). The use of this cryptic donor splice site would result in a transcript lacking the last 149 (not divisible by 3) nucleotides (barred) of exon 6. **Supplementary Figure 4**. SpliceAI-predicted impact of the *LPL* c.1160_1161insT variant on splicing. (a) SpliceAI predictions. (b) Explanation of the predicted results in the context of the wild-type *LPL* exon 8 and flanking intronic sequences. Exon 8 sequence is shown in upper case letters whereas intronic sequence is shown in lower case. The physiological obligate splice site acceptor and donor dinucleotides (ag and gt) are highlighted in blue. The *LPL* c.1160_1161insT variant was predicted to activate a downstream cryptic acceptor splice site (highlighted in red). The use of this cryptic acceptor splice site would result in a transcript lacking the first 47 (not divisible by 3) nucleotides of exon 8.

## Data Availability

All supporting data are available within the article or its supplemental material.

## Abbreviations

AP: Acute pancreatitis
APOA5: Apolipoprotein A5
APOC2: Apolipoprotein C2
FCS: Familial chylomicronemia syndrome
gnomAD: The Genome Aggregation Database
GPIHBP1: Glycosylphosphatidylinositol-anchored high density lipoprotein-binding protein 1
HGMD: Human Gene Mutation Database
HGVS: Human Genome Variation Society
HTG: Hypertriglyceridemia
HTG-AP: Hypertriglyceridemia-related acute pancreatitis
LMF1: Lipase maturation factor 1
LPL: Lipoprotein lipase
LUMC: Leiden University Medical Center
NI: Not informative
PCR: Polymerase chain reaction
TG: Triglyceride
