# Supplementary material for: Frameshift coding sequence variants in the LPL gene: identification of two novel events and exploration of the genotype–phenotype relationship for variants reported to date
Source: Lipids Health Dis. 2023 Aug 11;22:128. doi: 10.1186/s12944-023-01898-w (PMC10422730; doi:10.1186/s12944-023-01898-w)
Supplement: Supplementary file 1 — Additional file 1: Supplementary Table 1. Variants with an allele frequency of ≥1% found in the three patients. Supplementary Figure 1. Illustration showing that c.247_249+1del (in accordance with Human Genome Variation Society (HGVS) recommendations) may be alternatively described as c.246_249del. Exon 2 sequence is shown in upper case letters whereas intron 2 sequence is shown in lower case. The canonical 5’ splice site gt dinucleotide is highlighted in bold and blue. The deleted four nucleotides in each nomenclature version are barred. Supplementary Figure 2. SpliceAI predictions for the 55 LPL frameshift coding variants reported to date. Supplementary Figure 3. SpliceAI-predicted impact of the LPL c.899_921dup variant on splicing. (a) SpliceAI predictions. (b) Explanation of the predicted results in the context of the wild-type LPL exon 6 and flanking intronic sequences. Exon 6 sequence is shown in upper case letters whereas intronic sequence is shown in lower case. The physiological obligate acceptor and donor dinucleotides (ag and gt) are highlighted in blue. The 23-bp duplicated sequence is highlighted in red. The LPL c.899_921dup variant was predicted to activate an upstream cryptic donor splice site (highlighted in blue and underlined). The use of this cryptic donor splice site would result in a transcript lacking the last 149 (not divisible by 3) nucleotides (barred) of exon 6. Supplementary Figure 4. SpliceAI-predicted impact of the LPL c.1160_1161insT variant on splicing. (a) SpliceAI predictions. (b) Explanation of the predicted results in the context of the wild-type LPL exon 8 and flanking intronic sequences. Exon 8 sequence is shown in upper case letters whereas intronic sequence is shown in lower case. The physiological obligate splice site acceptor and donor dinucleotides (ag and gt) are highlighted in blue. The LPL c.1160_1161insT variant was predicted to activate a downstream cryptic acceptor splice site (highlighted in red). The use of this cryp [file 12944_2023_1898_MOESM1_ESM.docx]

**Supplementary Table 1.** Variants with an allele frequency of ≥1% found in the three patients

| Patient | Gene | rs number | mRNA reference accession | c.nomenclature | p.nomenclature | allele frequency in gnomAD (version v2.1.1) | | Clinical significance (as annotated by gnomAD) |
| --- | --- | --- | --- | --- | --- | --- | --- | --- |
|  |  |  |  |  |  | All populations | East Asians |  |
| #1 | *GPIHBP1* | rs56046179 | NM_178172.6 | c.295+27C>T |  | 0.1240 | 0.3010 | Benign |
|  | *APOA5* | rs2072560 | NM_001166598.2 | c.162-43A>G |  | 0.9000 | 0.7852 | Benign |
|  | *LMF1* | rs3751667 | NM_022773.4 | c.306G>A | p.Thr102= | 0.2773 | 0.4060 | Benign |
|  | *LMF1* | rs2277893 | NM_022773.4 | c.543G>A | p.Gly181= | 0.2640 | 0.3155 | Benign |
|  | *LMF1* | rs4984706 | NM_022773.4 | c.664-58G>C |  | 0.3549 | 0.4001 | Benign |
|  | *LMF1* | rs4984705 | NM_022773.4 | c.664-35T>C |  | 0.3607 | 0.4032 | Benign |
| #2 | *LPL* | rs343 | NM_000237.3 | c.430-34C>A |  | 0.0956 | 0.1501 | Benign |
|  | *LPL* | rs328 | NM_000237.3 | c.1421C>G | p.Ser474Ter | 0.0916 | 0.1117 | Benign |
|  | *LPL* | rs11570891 | NM_000237.3 | c.1428-11C>T |  | 0.0950 | 0.1114 | Not annotated |
|  | *APOA5* | rs2072560 | NM_001166598.2 | c.162-43A>G |  | 0.9000 | 0.7852 | Benign |
| #3 | *GPIHBP1* | rs11538389 | NM_178172.6 | c.41G>T | p.Cys14Phe | 0.1231 | 0.2998 | Benign |
|  | *APOA5* | rs2072560 | NM_001166598.2 | c.162-43A>G |  | 0.9000 | 0.7852 | Benign |
|  | *APOA5* | rs3135507 | NM_001166598.2 | c.457G>A | p.Val153Met | 0.0509 | 0.1192 | Benign |
|  | *LMF1* | rs3751666 | NM_022773.4 | c.194-28T>C |  | 0.4489 | 0.4008 | Benign |
|  | *LMF1* | rs3751667 | NM_022773.4 | c.306G>A | p.Thr102= | 0.2773 | 0.4060 | Benign |
|  | *LMF1* | rs2277892 | NM_022773.4 | c.540G>A | p.Thr180= | 0.1704 | 0.2488 | Benign |
|  | *LMF1* | rs2277893 | NM_022773.4 | c.543G>A | p.Gly181= | 0.2640 | 0.3155 | Benign |
|  | *LMF1* | rs4984706 | NM_022773.4 | c.664-58G>C |  | 0.3549 | 0.4001 | Benign |
|  | *LMF1* | rs4984705 | NM_022773.4 | c.664-35T>C |  | 0.3607 | 0.4032 | Benign |
|  | *LMF1* | rs4984948 | NM_022773.4 | c.1685C>G | p.Pro562Arg | 0.0435 | 0.1389 | Benign |


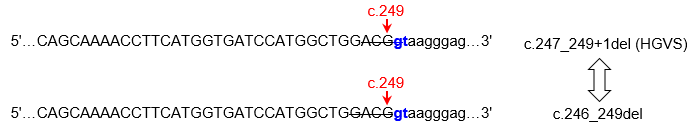


**Supplementary Figure 1.** Illustration showing that c.247_249+1del (in accordance with Human Genome Variation Society (HGVS) recommendations) may be alternatively described as c.246_249del. Exon 2 sequence is shown in upper case letters whereas intron 2 sequence is shown in lower case. The canonical 5’ splice site gt dinucleotide is highlighted in bold and blue. The deleted four nucleotides in each nomenclature version are barred.

c.10_11insTTCG


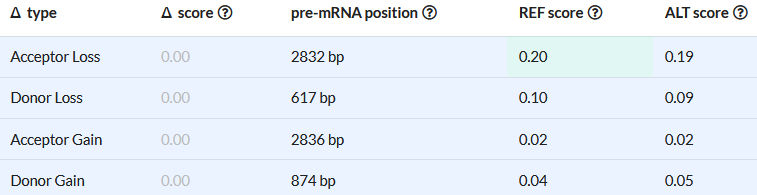


c.32dup


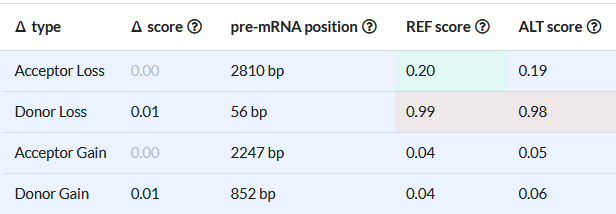


c.46_47del


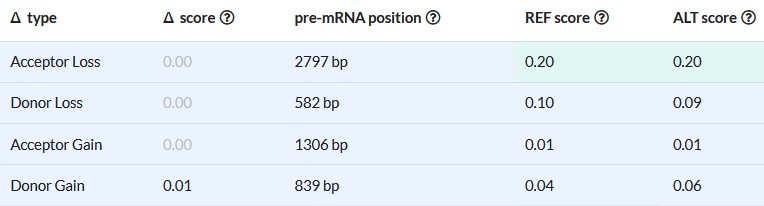


c.77_78+1del (used c.76_88 for prediction)


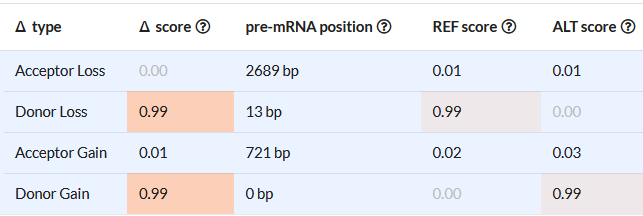


c.94_98del


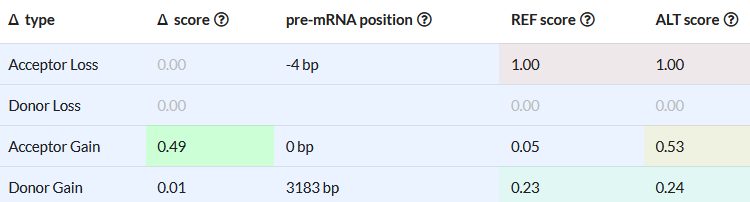


c.128dup


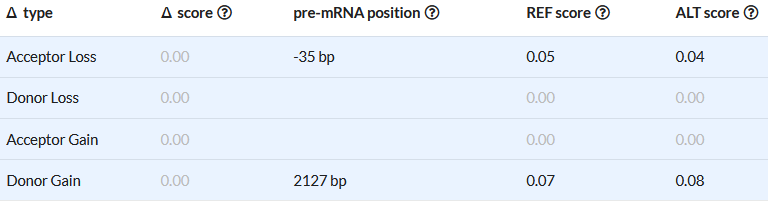


c.133_143del


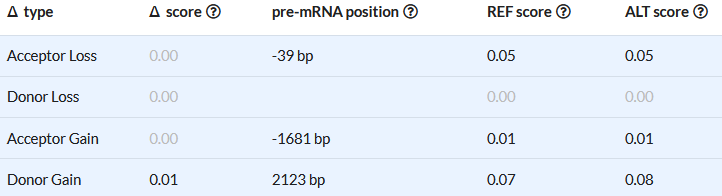


**Supplementary Figure 2.** SpliceAI predictions for the 55 *LPL* frameshift coding variants reported to date.

(*to be continued*)

c.133dup


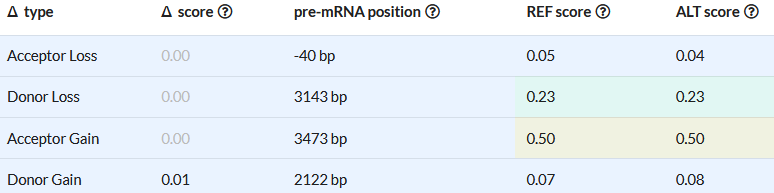


c.183dup


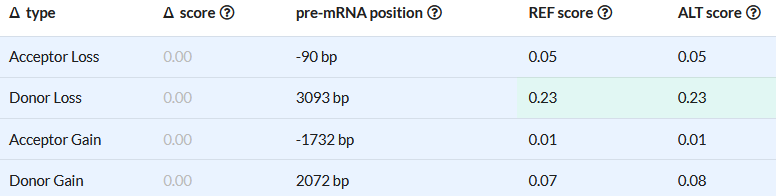


c.247_249+1del (used c.246_249del for prediction)


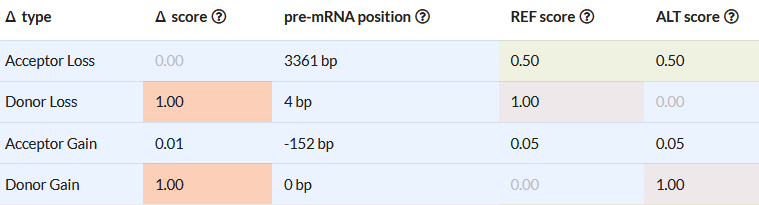


c.287_288del


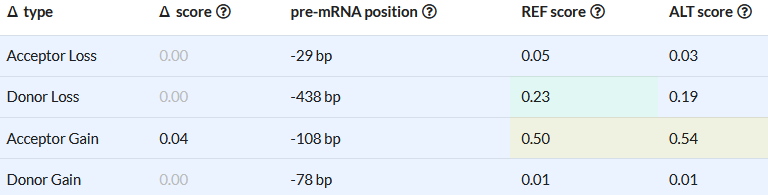


c.289_294delinsTTTGCCAAAA


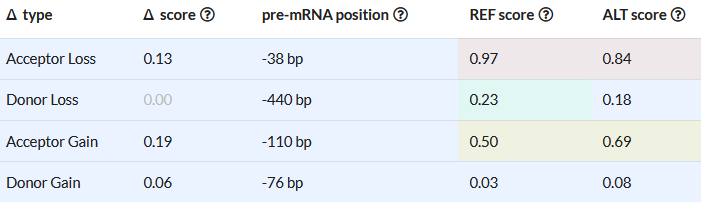


c.290_293delinsGG


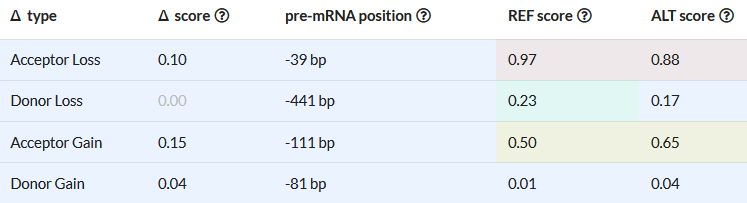


c.312del


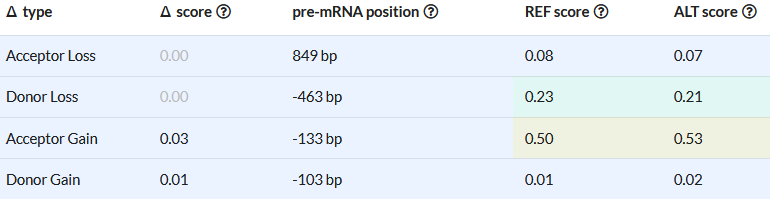


**Supplementary Figure 2** (*continued*)

c.334dup


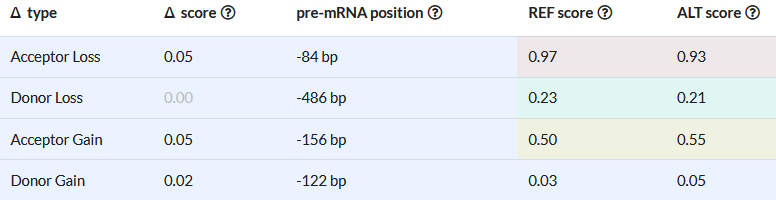


c.338_339insAGAGTACCATTCGATAC


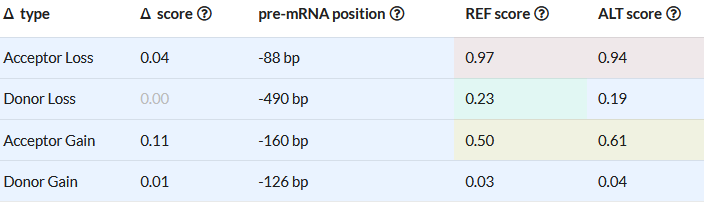


c.348_349insAGTACCATTCGACAGTC


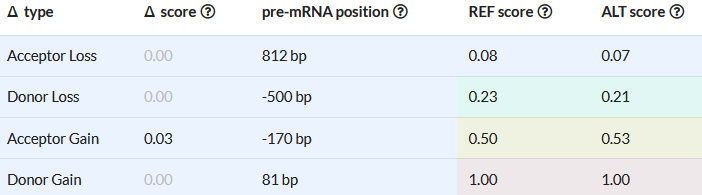


c.373dup


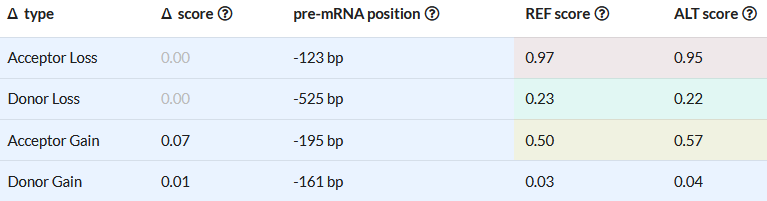


c.377_378insAGAGTACCATT


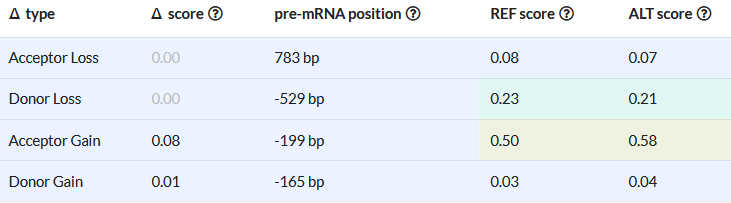


c.384delinsTGGGCT


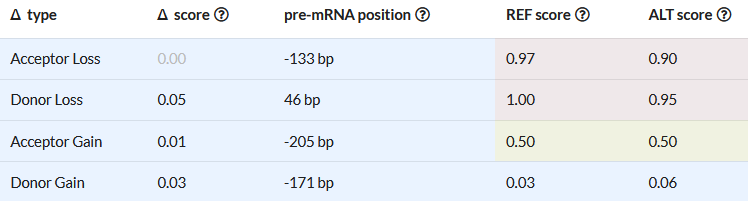


c.386_390del


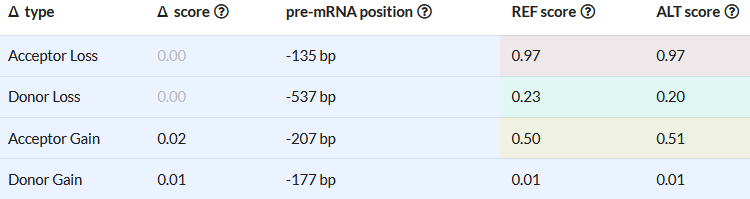


**Supplementary Figure 2** (*continued*)

c.431_432AG[3] (used c.431_432insGAGA for prediction)


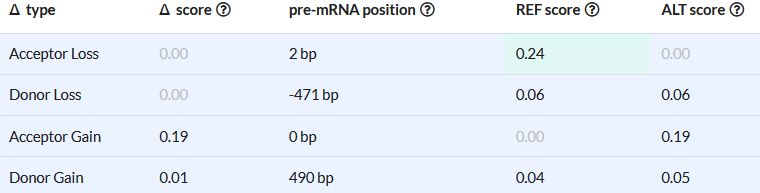


c.438del


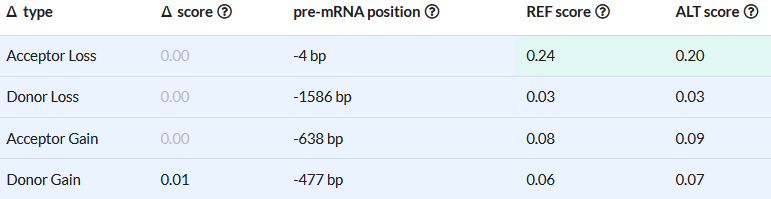


c.440_443del


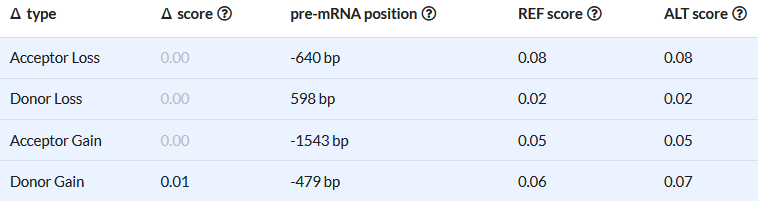


c.483delA


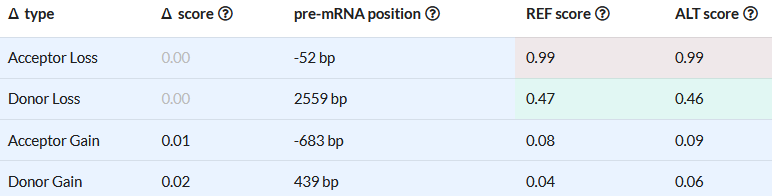


c.501_502insGAGAGTACCATTCGAGA


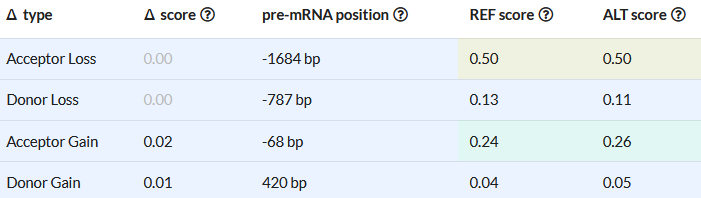


c.596del


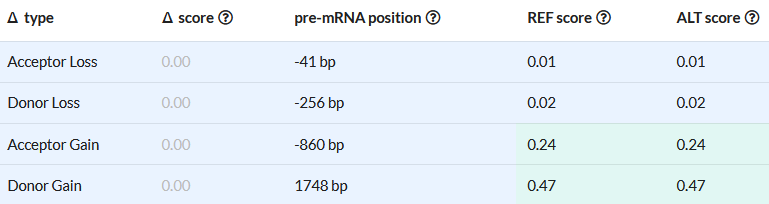


c.599del


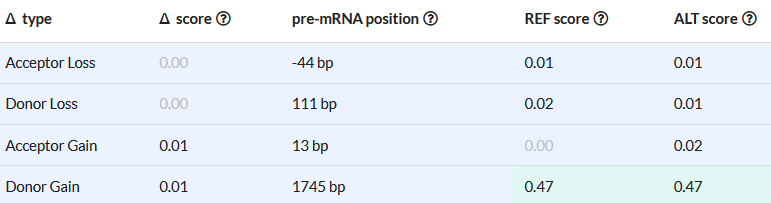


**Supplementary Figure 2** (*continued*)

c.624del


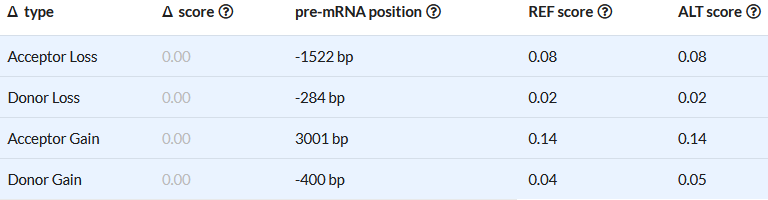


c.651del


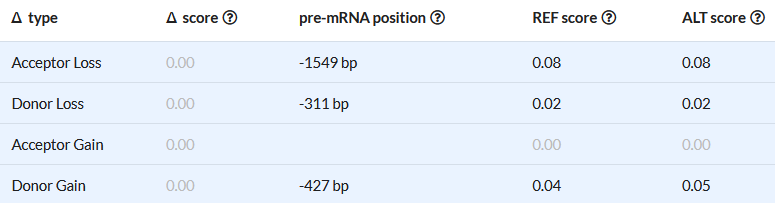


c.708del


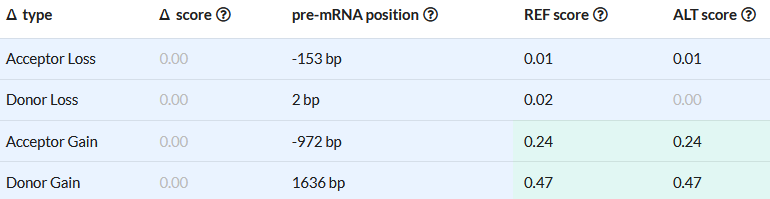


c.742del


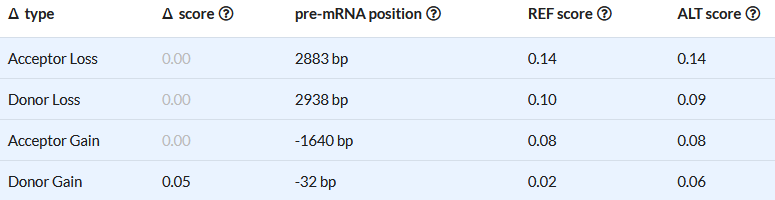


c.765_766del


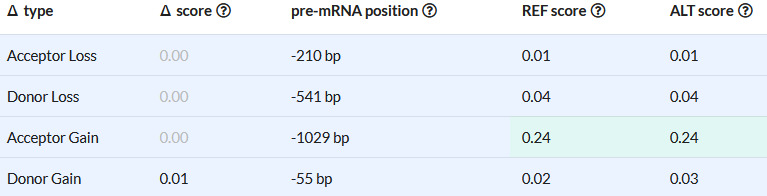


c.767_768insTAAATATT


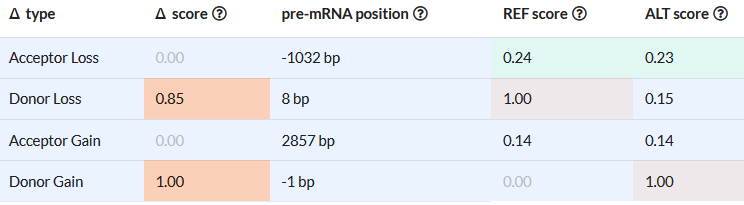


c.769_770insCA


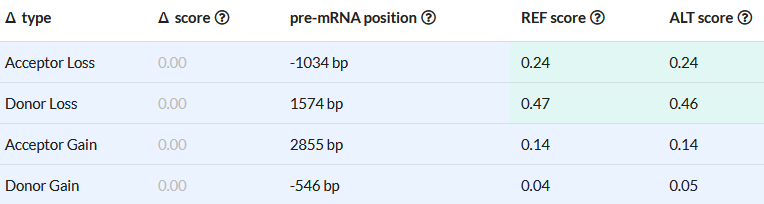


**Supplementary Figure 2** (*continued*)

c.835_836del


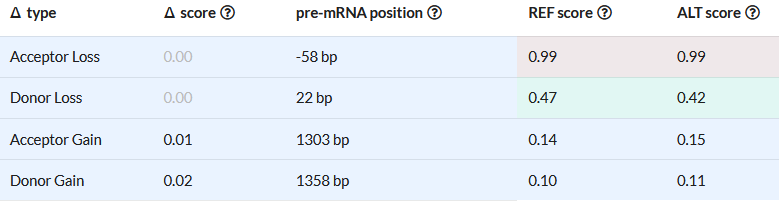


c.840del


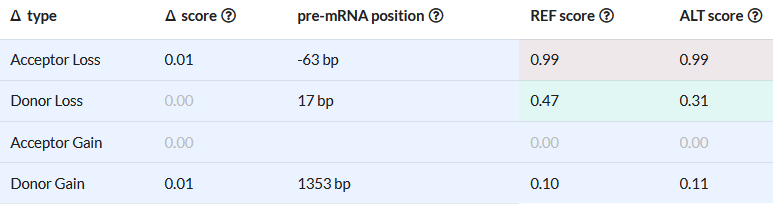


c.899_921dup


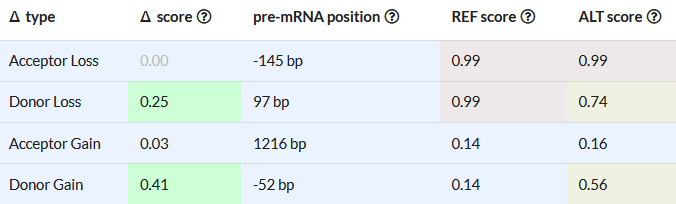


c.901del


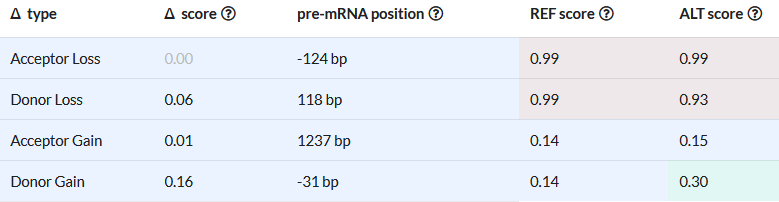


c.953del


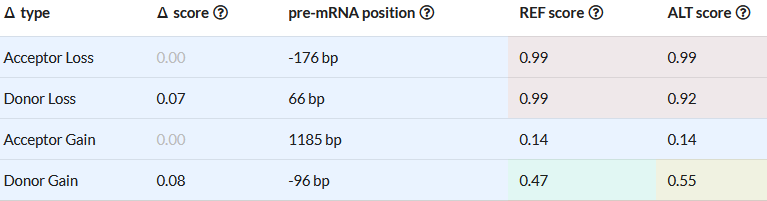


c.1008del


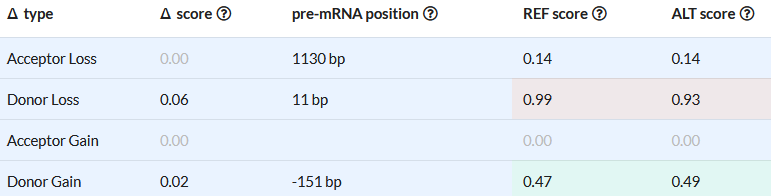


c.1010_1011insATTCGAGAGC


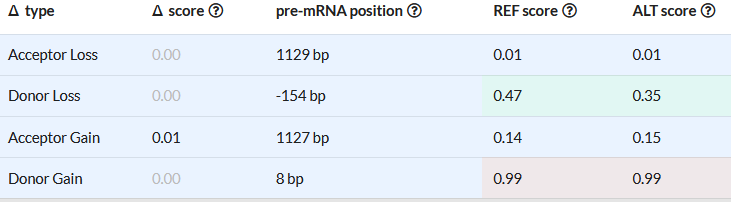


**Supplementary Figure 2** (*continued*)

c.1016_1017insC


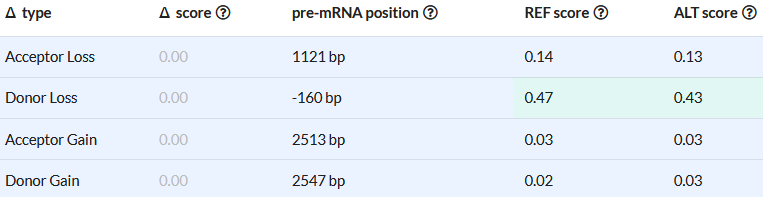


c.1044_1050del


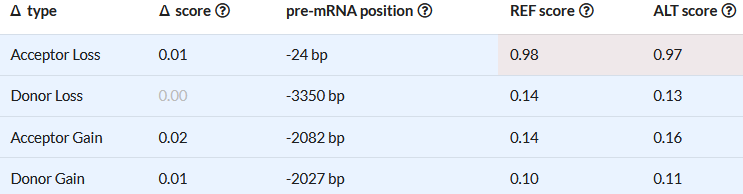


c.1081_1082insAGTA


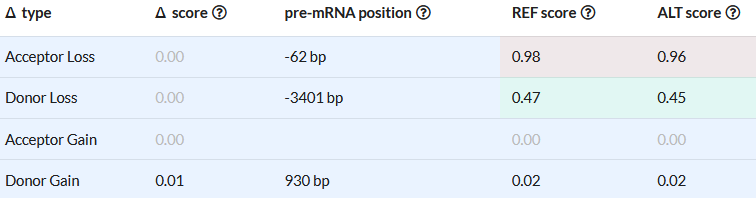


c.1107_1108insATTCGAAGAGCGC


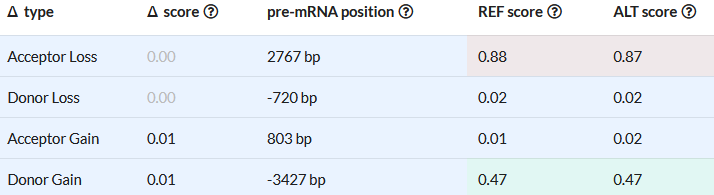


c.1115dup


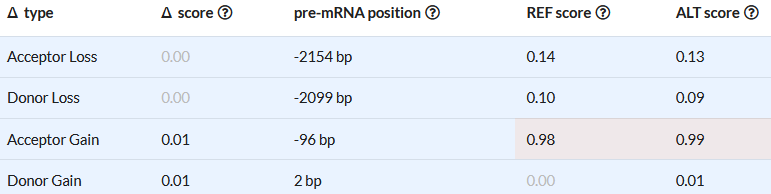


c.1119_1120insACCATTC


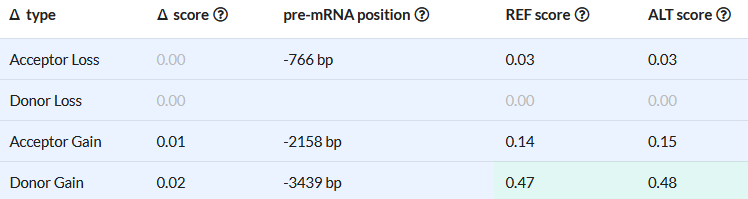


c.1121_1122insAGAGCGC


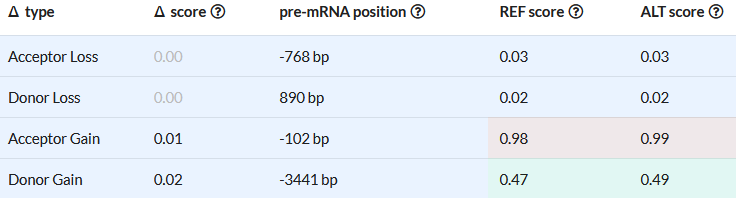


**Supplementary Figure 2** (*continued*)

c.1138_1139del


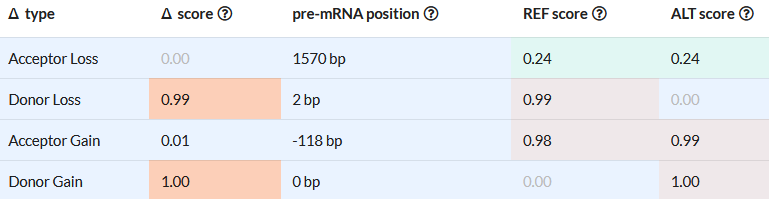


c.1160_1161insT


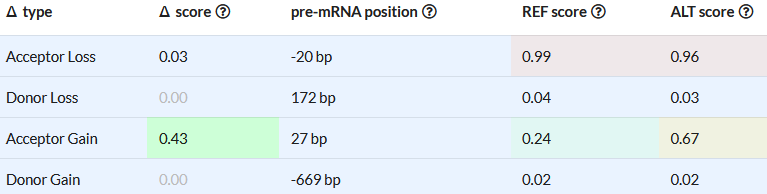


c.1163_1164insA


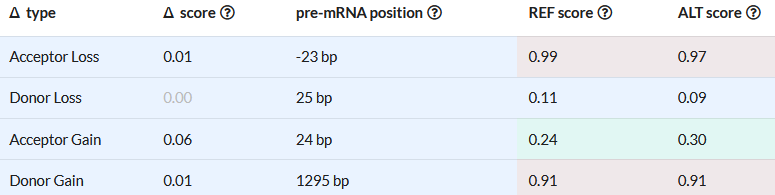


c.1303_1304del


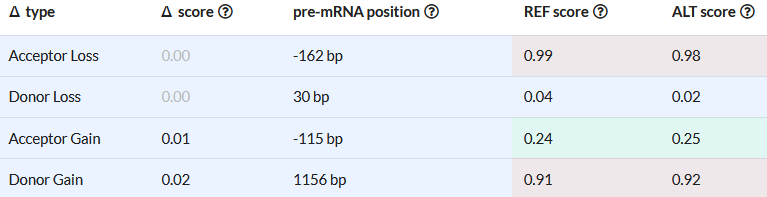


c.1306_1307insAGTACCATTC


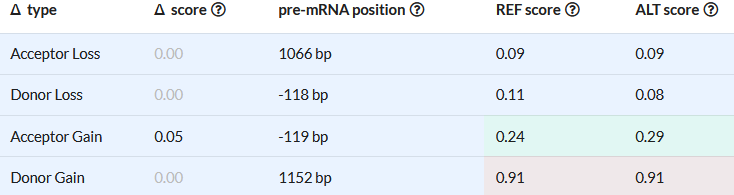


c.1373del


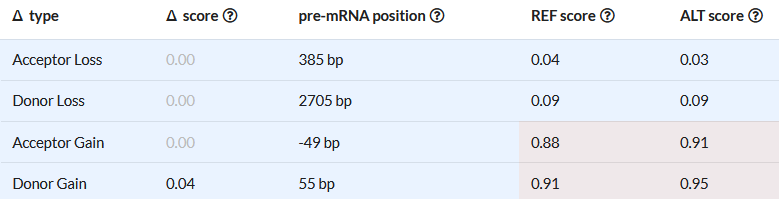


**Supplementary Figure 2** (*continued*)


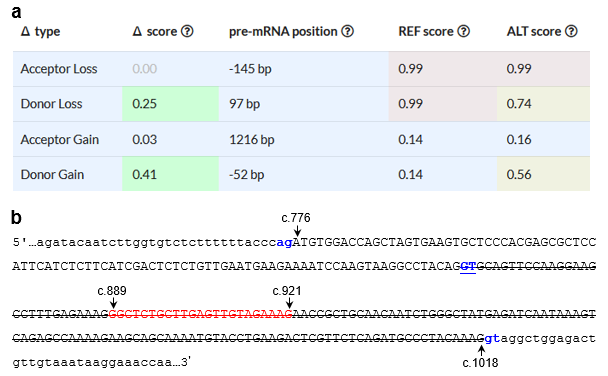


**Supplementary Figure 3. SpliceAI-predicted impact of the *LPL* c.899_921dup variant on splicing.** (**a**) SpliceAI predictions. (**b**) Explanation of the predicted results in the context of the wild-type *LPL* exon 6 and flanking intronic sequences. Exon 6 sequence is shown in upper case letters whereas intronic sequence is shown in lower case. The physiological obligate acceptor and donor dinucleotides (ag and gt) are highlighted in blue. The 23-bp duplicated sequence is highlighted in red. The *LPL* c.899_921dup variant was predicted to activate an upstream cryptic donor splice site (highlighted in blue and underlined). The use of this cryptic donor splice site would result in a transcript lacking the last 149 (not divisible by 3) nucleotides (barred) of exon 6.


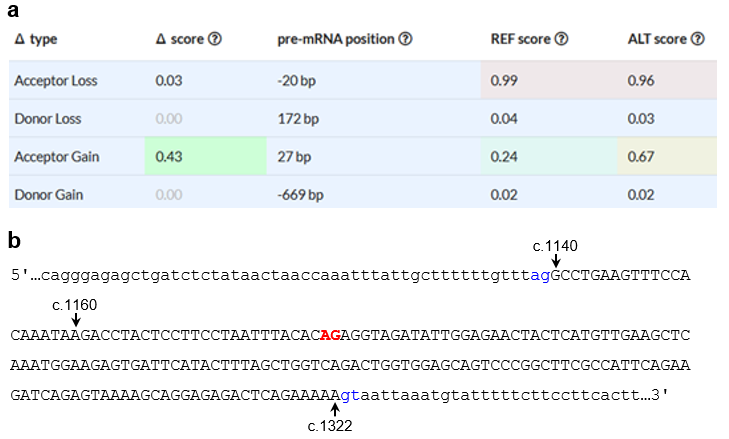


**Supplementary Figure 4. SpliceAI-predicted impact of the *LPL* c.1160_1161insT variant on splicing.** (**a**) SpliceAI predictions. (**b**) Explanation of the predicted results in the context of the wild-type *LPL* exon 8 and flanking intronic sequences. Exon 8 sequence is shown in upper case letters whereas intronic sequence is shown in lower case. The physiological obligate splice site acceptor and donor dinucleotides (ag and gt) are highlighted in blue. The *LPL* c.1160_1161insT variant was predicted to activate a downstream cryptic acceptor splice site (highlighted in red). The use of this cryptic acceptor splice site would result in a transcript lacking the first 47 (not divisible by 3) nucleotides of exon 8.
